# AI-Based Patient Safety Incident Reporting System for Nurses (SAFE-Nurse): Development and Validation Study

**DOI:** 10.2196/91321

**Published:** 2026-10-23

**Authors:** Nindya Febriyani Putri, Rini Rachmawaty, Nuurhidayat Jafar

**Affiliations:** 1 Nursing Management & Healthcare Quality Research Group, Faculty of Nursing, Hasanuddin University, Jl. Perintis Kemerdekaan KM. 10, Makassar, 90245, Indonesia, 62 81213916730; 2 Department of Nursing Management, Faculty of Nursing, Hasanuddin University, Makassar, Indonesia; 3 Department of Community Nursing, Faculty of Nursing, Hasanuddin University, Makassar, Indonesia

**Keywords:** artificial intelligence, digital health, incident reporting, nursing practice, patient safety, validation studies

## Abstract

This research letter describes the development and initial validation of SAFE-Nurse, an artificial intelligence–assisted patient safety incident reporting system for nurses, which demonstrated excellent content and face validity (average scale-level content validity index [S-CVI/Ave] 0.99; average scale-level face validity index [S-FVI/Ave] 1.00) and is ready to progress to usability testing.

## Introduction

Patient safety incident reporting is essential for health care quality, yet underreporting remains common due to workload, limited feedback, and blame culture among nurses [1,2]. Paper-based reporting further limits reporting efficiency and data management [3]. Although AI has potential to support healthcare processes [4], its application in patient safety incident reporting remains limited.

To address this gap, we developed Smart Assisted Feedback and Education for Nurses on Reporting Safety Events (SAFE-Nurse), an AI-assisted incident reporting system. This study evaluated its content and face validity.

## Methods

### Study Design

This research and development study used the ADDIE framework to develop SAFE-Nurse. The process comprised needs analysis (Analyze), system design (Design), prototype development and content and face validity assessment (Develop), pilot implementation (Implement), and usability evaluation using the System Usability Scale (Evaluate). This report focuses on the Develop phase.

### System Description (SAFE-Nurse)

SAFE-Nurse is an AI-assisted web application for patient safety incident reporting. It uses OpenAI Whisper (whisper-1) for voice-to-text transcription and GPT-4o-mini via an application programming interface (API) to normalize incident narratives, recommend incident categories, and assess chronology completeness. Severity grading is calculated deterministically by the backend using predefined impact and probability scores. AI outputs are reviewed and confirmed by nurses and may be revised by authorized reviewers. Detailed information on the AI pipeline, API inputs and outputs, prompt structure, and severity-grading process is provided in Multimedia Appendix 1.

The system supports 3 roles: staff nurses, head nurses, and quality and patient safety personnel. Staff nurses submit reports and receive AI-assisted guidance. Head nurses review, validate, revise, or reject reports and review AI-generated classifications. Quality and patient safety personnel perform final verification and monitor incidents through the dashboard (Figure 1).

**Figure 1.**
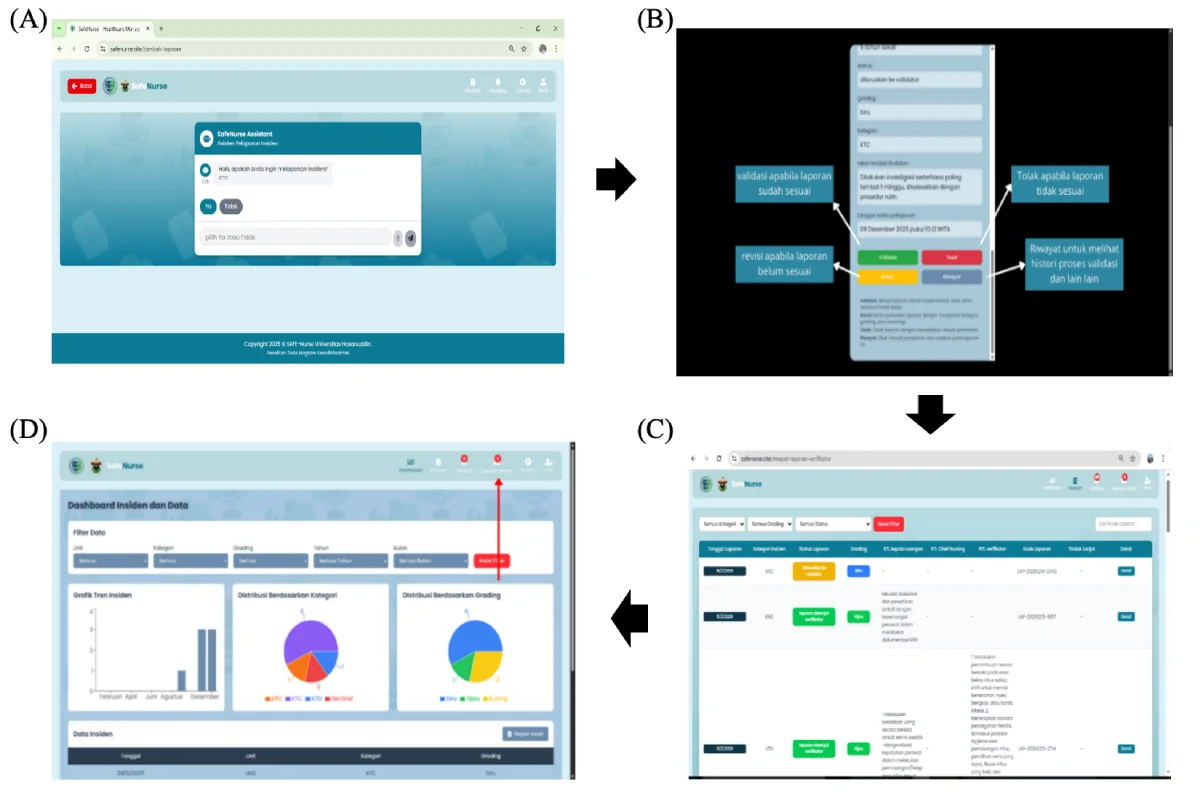
SAFE-Nurse interfaces: (A) AI-assisted incident reporting interface for staff nurses; (B) validation interface for head nurses; (C) incident reporting dashboard; (D) analytical dashboard for quality and patient safety monitoring.

### Study Setting and Participants

Content validity was assessed by 6 experts selected purposively through professional and institutional networks in Makassar and Yogyakarta, Indonesia. Eligible experts had at least 5 years of hospital experience and expertise in nursing informatics, nursing management, or patient safety and quality, with 2 experts from each field. Assessments were conducted at their respective institutions or remotely via Zoom. Experts were invited via email or WhatsApp.

Face validity was assessed among 17 potential SAFE-Nurse users at RSIA Paramount Makassar, Indonesia, selected purposively using maximum variation sampling. Participants included staff nurses (n=7), head nurses (n=7), and quality and patient safety personnel (n=3), recruited through hospital contacts based on their patient safety reporting roles. The content- and face-validity groups were separate and nonoverlapping.

### Instrument

A researcher-developed 43-item questionnaire based on SAFE-Nurse system features and study objectives assessed content and face validity (Multimedia Appendix 2). It comprised 5 domains: content relevance and objectives (7 items), clarity and usability (10 items), functionality and workflow (7 items), technical aspects and confidentiality (9 items), and usefulness and impact (10 items). Content validity used a 4-point Likert scale; face validity used a dichotomous “clear”/“unclear” scale with optional comments. All 43 items were retained. The questionnaire was not pretested or pilot-tested.

### Validity Procedure (Content and Face Validity)

Participants received study information and access credentials to explore SAFE-Nurse before completing the questionnaire during scheduled in-person sessions. Experts assessed system relevance, whereas end users assessed clarity after interacting with SAFE-Nurse. Participants could also provide comments or suggestions in the open-ended section using the prompt: “Please provide comments or suggestions regarding aspects of the SAFE-Nurse system that require improvement.”

### Data Analysis

Data were analyzed descriptively. Content and face validity were assessed using the content validity index (CVI) and face validity index (FVI), respectively. Open-ended comments were summarized by refinement area.

### Ethical Considerations

The study was approved by the Hasanuddin University Research Ethics Committee (No. 659/UN4.18.3./TP.01.02/2025). Written informed consent was obtained, and data were anonymized and kept confidential.

## Results

### Content and Face Validity

Table 1 summarizes the results. Among 6 experts, item-level CVI ranged from 0.83 to 1.00; scale-level average and universal agreement CVI were 0.99 and 0.93, respectively. Among 17 end users, item-level FVI ranged from 0.94 to 1.00; scale-level average and universal agreement FVI were 1.00 and 0.98, respectively. Overall, SAFE-Nurse showed strong agreement regarding relevance and clarity.

**Table 1.** Content validity index (CVI) and face validity index (FVI) of the SAFE-Nurse system.

Domain	Number of items	I-CVI^a^	I-FVI^b^
Content relevance and objectives	7	1.00	1.00
Clarity and usability	10	1.00	1.00
Functionality and workflow	7	1.00	1.00
Technical and confidentiality	9	1.00	1.00
Usefulness and impact	10	0.83‐1.00	0.94‐1.00
Scale average (S-CVI^c^ and S-FVI^d^)	—^e^	0.99	1.00
Universal agreement (S-CVI and S-FVI)	—	0.93	0.98

a I-CVI: item-level content validity index.

b I-FVI: item-level face validity index.

c S-CVI: scale-level content validity index.

d S-FVI: scale-level face validity index.

e Not applicable.

### Expert Feedback and System Refinement

Expert comments identified areas for refinement in workflow, interface design, reporting, and monitoring. Refinements included reorganizing incident categories and severity grading, simplifying data entry, adding progress indicators, role-specific guidance, notification reminders, and follow-up fields. Integration with the hospital information system (SIMRS) was also recommended.

## Discussion

### Principal Findings

SAFE-Nurse demonstrated excellent content and face validity, indicating strong consensus among experts and end users regarding its relevance and clarity for patient safety incident reporting [5-7]. These findings support system refinement and progression to usability testing but do not establish reliability, real-world usability, or clinical effectiveness.

The findings are consistent with research demonstrating the potential of AI for patient safety event classification and human-AI collaboration [8]. Generative AI has also been explored for critical incident analysis, although further evaluation is needed before broader application [9]. Similar nurse-led AI work has used expert review and iterative refinement to improve the structure and completeness of patient safety incident reports [10]. Consistent with these approaches, AI-generated classifications and chronology feedback in SAFE-Nurse are intended to support, rather than replace, human review.

This study has three limitations. First, the expert and end-user groups were relatively small. Second, the 43-item questionnaire was not pretested or pilot-tested, so potential issues related to comprehensibility or respondent burden may not have been identified. Third, clinical performance was not assessed.

### Conclusion

SAFE-Nurse demonstrated excellent content and face validity as an AI-assisted incident reporting system. These findings indicate strong consensus regarding the relevance and clarity of SAFE-Nurse and support progression to usability testing; they indicate that SAFE-Nurse is relevant and appropriate for Indonesian hospitals.

## Supplementary material

10.2196/91321 Multimedia Appendix 1 Technical description of the SAFE-Nurse AI pipeline.

10.2196/91321 Multimedia Appendix 2 Content and face validity evaluation instrument for the SAFE-Nurse system.

## Abbreviations

ADDIE: Analyze, Design, Develop, Implement, and Evaluate
API: application programming interface
CVI: content validity index
FVI: face validity index
SAFE-Nurse: Smart Assisted Feedback and Education for Nurses on Reporting Safety Events
