# Supplementary material for: AI-Based Patient Safety Incident Reporting System for Nurses (SAFE-Nurse): Development and Validation Study
Source: JMIR Form Res. 2026 Oct 23;10:e91321. doi: 10.2196/91321 (PMC13727391; doi:10.2196/91321)
Supplement: Supplementary file 1 [file formative-v10-e91321-s001.docx]

**Technical Description of the SAFE-Nurse AI Pipeline**

A.1 Overview of the SAFE-Nurse AI Pipeline

SAFE-Nurse is an AI-assisted patient safety incident reporting application designed to support nurses in preparing and reviewing incident reports. The system does not make autonomous final decisions. AI-generated outputs are reviewed and confirmed by nurses and may be revised by authorized hospital personnel. The main workflow is as follows:

1. The nurse enters incident information manually or uses voice input.
2. For voice input, OpenAI Whisper (whisper-1) converts Indonesian speech into text.
3. The backend sends selected report fields to GPT-4o-mini for text normalization and initial incident-category recommendation.
4. The nurse reviews and confirms the AI-generated output.
5. GPT-4o-mini also assesses whether the incident chronology is complete and clear.
6. The backend calculates the severity score and grade using predefined impact and probability rules.
7. The completed report is stored and routed to the appropriate personnel for review.

GPT-4o-mini is accessed from the backend using the OpenAI Software Development Kit (SDK). The API key is stored in the server environment and is not exposed to the client application.

A.2 Data Sent to GPT-4o-mini

The incident-classification endpoint is /api/laporan/clean and can only be accessed by authenticated users with the nurse role. Before the AI request is made, the backend validates the required report fields, including patient information, incident information, chronology, initial actions, impact, and probability. However, only selected fields are transmitted to GPT-4o-mini for classification and normalization:

1. Reporting Unit;
2. Incident Location;
3. Incident Title;
4. Incident Chronology;
5. Initial Action; and
6. Impact.

Patient name, medical record number, age, sex, admission date, incident date, reported party, action performer, and probability are not included in the GPT-4o-mini classification request. These data remain processed by the backend and are recombined with the AI response after processing.

The user prompt is generated as a JSON object containing only the selected fields. The model therefore receives only a restricted subset of the incident-report data rather than the complete report.

A.3 Prompt Structure and API Configuration

The backend sends two messages to the Chat Completions API:

1. A system prompt defining the model's role, text-normalization tasks, incident-classification rules, and required output format; and
2. A user prompt containing the selected incident-report data in JSON format.

The GPT-4o-mini request uses the following configuration:

1. Model	: gpt-4o-mini
2. Temperature	: 0
3. Response format	: JSON object

The system prompt instructs GPT-4o-mini to normalize capitalization, spelling, and spacing in the reporting unit, incident location, incident title, chronology, and initial action. It also instructs the model to assign one of five incident categories based on the information provided.

The expected output is a JSON object containing the normalized reporting unit, incident location, incident title, chronology, initial action, and recommended incident category. The system does not use a confidence score or a hospital-specific model trained on local data.

A.4 Incident Category Recommendation

GPT-4o-mini provides an initial incident-category recommendation according to predefined rules included in the system prompt:

| **Incident condition** | **Recommended category** |
| --- | --- |
| Incident has not occurred and remains a potential event | KPC |
| Incident occurred but was prevented before reaching the patient | KNC |
| Incident reached the patient without causing injury | KTC |
| Incident caused mild or moderate injury | KTD |
| Incident caused severe injury or death | Sentinel |

The category generated by GPT-4o-mini is an initial recommendation rather than a final determination. The nurse reviews the recommendation before submitting the report. Head nurses, chief nursing personnel, or quality and patient safety verifiers can subsequently approve, reject, or request revisions. Changes to the category, chronology, or severity grade are recorded in the system history.

A.5 Chronology Completeness Assessment

SAFE-Nurse includes a separate endpoint, /api/laporan/validateChronology, for assessing the completeness and clarity of the incident chronology.

The endpoint receives the incident chronology and incident title and sends them to GPT-4o-mini. The system prompt instructs the model to assess the chronology according to the following elements:

1. Who	: persons involved;
2. What	: the action or event that occurred;
3. When	: the date and time of the event;
4. Where	: the location of the event; and
5. How	: the sequence or mechanism of the event.

The model returns whether the chronology is complete, an evaluation of the chronology, and the missing elements. The AI output provides feedback only and does not automatically modify the chronology.

A.6 Severity Grading and Follow-up Recommendations

Severity grading is not calculated by GPT-4o-mini. Instead, the backend applies a deterministic impact-probability matrix.

**Impact is scored from 1 to 5:**

| **Impact** | **Score** |
| --- | --- |
| No injury | 1 |
| Mild injury | 2 |
| Moderate injury | 3 |
| Severe injury | 4 |
| Death | 5 |

**Probability is also scored from 1 to 5:**

| **Probability** | **Score** |
| --- | --- |
| Once every 5 years | 1 |
| Once every 2–5 years | 2 |
| Once every 1–2 years | 3 |
| Several times per year | 4 |
| Monthly or weekly | 5 |

**The grading score is calculated as:**

Severity grade score = Impact score × Probability score

**The resulting score is mapped to the following grades:**

| **Score** | **Grade** | **System recommendation** |
| --- | --- | --- |
| 1-4 | Blue | Simple investigation and routine management |
| 5-9 | Green | Simple investigation and risk-impact review |
| 10-14 | Yellow | Root cause analysis (RCA), immediate action, and senior management attention |
| 15-25 | Red | Root cause analysis (RCA), immediate action, and director-level attention |

Because these rules are deterministic, the same impact and probability inputs produce the same severity grade and follow-up recommendation. The backend stores the impact score, probability score, grading score, grade, and recommendation with the incident report.

A.7 Report Submission, Storage, and Human Review

The /api/laporan/generate endpoint confirms that the required fields are available, verifies the nurse and unit information, calculates the severity score, and stores the report in Supabase.

The system routes reports according to their category and severity grade and sends notifications to relevant personnel. Head nurses, chief nursing personnel, and quality and patient safety verifiers can approve, reject, or revise reports. These actions are recorded in the system history to maintain traceability of changes and final decisions.

A.8 Summary of AI and Deterministic Functions

The SAFE-Nurse pipeline uses AI for three functions: voice transcription using Whisper-1, text normalization and initial incident-category recommendation using GPT-4o-mini, and chronology-completeness assessment using GPT-4o-mini.

GPT-4o-mini does not calculate severity grades. Severity grading and follow-up recommendations are generated deterministically by the backend using the predefined impact-probability matrix. All AI-generated outputs remain subject to nurse confirmation and authorized human review.
