# Supplementary material for: AI-Based Patient Safety Incident Reporting System for Nurses (SAFE-Nurse): Development and Validation Study
Source: JMIR Form Res. 2026 Oct 23;10:e91321. doi: 10.2196/91321 (PMC13727391; doi:10.2196/91321)
Supplement: Supplementary file 2 [file formative-v10-e91321-s002.docx]

**SAFE-Nurse Application Evaluation Form**

**(AI-Based Patient Safety Incident Reporting System)**

**Purpose:**

This questionnaire aims to evaluate the SAFE-Nurse application in supporting patient safety incident reporting among nurses. The evaluation covers aspects of relevance, clarity, appropriateness, and usability of the system based on hospital incident reporting practices.

**Instructions:**

This questionnaire is used for two types of evaluation:

1. Content Validity (for experts):

This evaluation assesses the relevance and appropriateness of each item in representing the SAFE-Nurse system.

Please use the following scale:

1 = Not relevant

2 = Somewhat relevant

3 = Quite relevant

4 = Highly relevant

2. Face Validity (for end users/nurses):

This evaluation assesses the clarity and comprehensibility of each item.

Please select one of the following:

1 = Clear

2 = Unclear

Please read each item carefully and provide your assessment based on your experience and professional judgment.

**Additional Notes:**

1. Please provide comments or suggestions for improvement in the space provided.
2. Your feedback is highly valuable for improving and refining the SAFE-Nurse system.

**Respondent Information:**

Name	: ....................................................

Field/Unit	: ....................................................

Position/Role	: ....................................................

Institution	: ....................................................

Highest Education	: ....................................................

Years of Experience	: ....................................................

Date	: ....................................................

Signature	: ....................................................

**Content and Face Validity Evaluation Form**

| **No** | **Item** | **Content Validity (1–4)** | **Face Validity (Clear/ Unclear)** | **Comments** |
| --- | --- | --- | --- | --- |
| A. Content and Objective Appropriateness | | | | |
| 1. | The purpose of the SAFE-Nurse application (to encourage incident reporting among nurses) is clearly presented on the initial interface. |  |  |  |
| 2. | The reporting workflow aligns with national incident reporting guidelines (Indonesian Ministry of Health Regulation No. 11 of 2017 and Hospital Accreditation Standards 2024). |  |  |  |
| 3. | The SAFE-Nurse Assistant feature clearly represents the incident reporting process and meets nurses’ needs. |  |  |  |
| 4. | The AI-based validation and classification/grading features support the incident management process by the head nurse. |  |  |  |
| 5. | The dashboard and analytical features clearly present incident reporting outcomes for quality teams. |  |  |  |
| 6. | The information and content presented are appropriate to hospital incident reporting context. |  |  |  |
| 7. | AI-generated classification and grading align with national patient safety guidelines. |  |  |  |
| B. Clarity and Usability | | | | |
| 8. | The interface and menu structure are easy to understand. |  |  |  |
| 9. | The language and terminology used are clear and unambiguous. |  |  |  |
| 10. | Instructions within the application are clear and helpful. |  |  |  |
| 11. | The SAFE-Nurse Assistant provides clear conversational guidance. |  |  |  |
| 12. | The voice input (voice-to-text) feature is easy to use and accurate. |  |  |  |
| 13. | The tutorial materials support user understanding. |  |  |  |
| 14. | The head nurse interface is easy to use for validation and grading. |  |  |  |
| 15. | Layout, icons, and colors support usability. |  |  |  |
| 16. | Navigation between pages is smooth and intuitive. |  |  |  |
| 17. | Text size and interface elements are readable on mobile devices. |  |  |  |
| C. Functionality and Workflow | | | | |
| 18. | The reporting workflow reflects a clear hierarchical process (nurse → head nurse → quality team). |  |  |  |
| 19. | Notification features clearly inform users about report status. |  |  |  |
| 20. | Validation features (approve, revise, reject) are easy to use. |  |  |  |
| 21. | AI-generated classification is understandable. |  |  |  |
| 22. | The dashboard presents reports clearly by time, status, and category. |  |  |  |
| 23. | Verification features are easy to use and clear. |  |  |  |
| 24. | Analytical outputs provide a comprehensive overview of reporting. |  |  |  |
| D. Technical and Confidentiality | | | | |
| 25. | Login and authentication processes are easy and reliable. |  |  |  |
| 26. | User access is appropriately restricted based on roles. |  |  |  |
| 27. | User profile information is clearly presented. |  |  |  |
| 28. | Reporter confidentiality is well protected. |  |  |  |
| 29. | Incident data are securely stored. |  |  |  |
| 30. | Security features (password, logout, access control) function properly. |  |  |  |
| 31. | The system operates stably without errors. |  |  |  |
| 32. | System response time is adequate. |  |  |  |
| 33 | Security indicators help users understand account safety. |  |  |  |
| E. Usefulness and Impact | | | | |
| 34. | The application simplifies incident reporting compared to manual methods. |  |  |  |
| 35. | The SAFE-Nurse Assistant improves reporting efficiency. |  |  |  |
| 36. | AI-based grading supports decision-making by head nurses. |  |  |  |
| 37. | Digital validation improves efficiency. |  |  |  |
| 38. | The dashboard supports quality monitoring. |  |  |  |
| 39. | Data visualization supports decision-making. |  |  |  |
| 40. | The system encourages reporting participation. |  |  |  |
| 41. | The system reduces reporting barriers. |  |  |  |
| 42. | The system supports patient safety culture. |  |  |  |
| 43. | The system is appropriate for implementation in hospitals. |  |  |  |
